# Editorial on the Special Issue “Genetic and Molecular Basis of Inherited Disorders”

**DOI:** 10.3390/genes15101259

**Published:** 2024-09-27

**Authors:** Rodolfo Iuliano, Francesco Paduano

**Affiliations:** 1 Medical Genetics Unit, Renato Dulbecco University Hospital, 88100 Catanzaro, Italy; 2 Department of Health Sciences, Campus S. Venuta, University Magna Graecia of Catanzaro, 88100 Catanzaro, Italy; 3 Stem Cells and Medical Genetics Units, Tecnologica Research Institute and Marrelli Health, 88900 Crotone, Italy

## Abstract

This Special Issue of *Genes*, titled “Genetic and Molecular Basis of Inherited Disorders”, presents a collection of pioneering research articles that advance our understanding of the genetic mechanisms underlying various hereditary diseases. The studies employ cutting-edge genomic techniques, including next-generation sequencing and genome-wide association studies, to elucidate novel genetic variants and their functional implications. Key investigations span a diverse range of conditions, from congenital idiopathic nystagmus and hereditary hearing loss to familial hypercholesterolemia and rare cancer predisposition syndromes. Notable findings include the identification of new gene–disease associations in congenital anomalies of the kidney and urinary tract, the discovery of large genomic rearrangements in breast cancer susceptibility, and insights into the genetic basis of pigmentary traits and associated disease risks. This Special Issue also highlights the significance of copy number variations and rare structural variants in disease pathogenesis. Collectively, these studies underscore the complexity of genetic variation in inherited disorders and demonstrate the critical role of integrating advanced genetic analyses with clinical practice to enhance diagnostic precision and develop targeted therapeutic approaches.

## 1. Editorial

The exploration of the genetic and molecular underpinnings of inherited disorders represents one of the most intriguing and challenging frontiers in contemporary biomedical research [1]. 

Driven by this exploration, the field of medical genetics has undergone significant transformation, evolving into an interdisciplinary domain where clinical genomics intersects with functional studies [2]. This evolution has been propelled by advances in next-generation sequencing (NGS) technologies, including exome and whole-genome analyses, which have been instrumental in uncovering the etiologies of various genetic disorders [3,4]. These technological breakthroughs have not only expanded our understanding of inherited conditions but have also revolutionized diagnostic approaches and therapeutic strategies [5]. For example, studies have demonstrated the utility of NGS in diagnosing rare genetic diseases and uncovering novel therapeutic targets [6,7].

This Special Issue of *Genes*, titled “Genetic and Molecular Basis of Inherited Disorders”, compiles a collection of pioneering research articles that substantially advance our understanding of the genetic mechanisms underlying a wide array of hereditary diseases. Through these contributions, we gain fresh insights into the pathophysiology of these conditions, paving the way for innovative approaches to treatment and prevention.

The featured articles emphasize the critical role of precision medicine and underscore the importance of integrating genetic research with clinical practice to improve patient outcomes. A recurring theme across these studies is the complexity of genetic variation and its impact on disease expression and treatment. For instance, the study by *Lazareva et al.* examines the shared characteristics of genes implicated in multiple rare diseases and the features of genetic variants that may elucidate the mechanisms behind phenotypic heterogeneity. Their findings reveal that while intragenic localization and variant type are more significant for genes linked to autosomal dominant diseases, these factors alone are insufficient to fully explain heterogeneous gene–disease relationships. This highlights the necessity of exploring additional factors that contribute to phenotypic heterogeneity [8].

In an effort to advance our understanding of the molecular mechanisms underlying genetic diseases, *Arshad et al.* conducted a study on congenital idiopathic nystagmus (CIN) within a consanguineous Pakistani family, identifying a novel mutation in the *FRMD7* gene. This mutation introduces a premature stop codon, resulting in a truncated and non-functional protein that disrupts neuronal development and eye movement control. The study not only broadens the mutational spectrum of the *FRMD7* gene but also deepens our understanding of the genetic mechanisms underlying CIN. Insights such as these are vital for improving diagnostic precision and developing targeted therapies for complex ocular disorders [9].

Further expanding our genetic understanding, *Farré et al.* conduct a comprehensive analysis of pigmentary traits and their association with various diseases in a large South European cohort. Through genome-wide association studies (GWASs) and polygenic risk scores (PRSs), the study identifies 37 risk loci and several genes, both known and novel, highlighting the strong correlation between fair skin phototype and an increased risk of skin diseases. By demonstrating the pleiotropy of pigmentary traits, this research underscores the importance of considering genetic backgrounds when assessing disease risk, thereby paving the way for more personalized approaches in dermatological and ocular healthcare [10].

Continuing this exploration of genetic complexity, Nascimento et al. investigate the genetic factors contributing to hereditary hearing loss by examining the DFNA58 locus. Their research identifies a genomic duplication involving three protein-coding genes, among which *CNRIP1* identified as a key player in the auditory system. Utilizing zebrafish and mouse models, the study demonstrates that *CNRIP1* is the most significant candidate gene influencing hearing, highlighting its potential as a target for future therapies aimed at treating hereditary hearing loss [11].

Transitioning to cancer susceptibility, *Ben Aissa-Haj et al.* explore large rearrangements in the *CDH1* gene among breast cancer patients negative for *BRCA1/2* mutations. By employing advanced genomic techniques, they identify significant structural variants linked to increased cancer risk, underscoring the potential role of *CDH1* as a critical factor in cancer predisposition beyond traditional genetic markers. Their findings highlight the importance of incorporating the screening of large rearrangements in the *CDH1* gene into the genetic testing of breast cancer cases [12].

Continuing with the theme of hereditary conditions, *Al-Hamed et al.* investigate congenital anomalies of the kidney and urinary tract (CAKUT) in two consanguineous families from Saudi Arabia. They discover novel homozygous missense variants in the *GFRA1* and *NPNT* genes associated with CAKUT, highlighting the importance of these genes in kidney development. Their research extends the genetic understanding of CAKUT and emphasizes the necessity of including these genes in genetic testing for kidney anomalies, offering new avenues for diagnosis and treatment [13].

In an effort to unravel the complexities of inherited disorders, *Rutkowska et al.* present a pioneering approach to diagnosing familial hypercholesterolemia (FH). By integrating next-generation sequencing (NGS) with advanced bioinformatics tools, their research aims at enhancing the detection of copy number variations (CNVs), a critical yet underexplored component of FH’s genetic landscape. This study not only uncovers previously undetected large-scale variations in the *LDLR* gene but also advocates for more comprehensive genetic screening methods. The findings pave the way for improved CNV screening in FH patients, highlighting the transformative potential of combining NGS data with bioinformatic tools in revolutionizing patient care [14].

In an effort to unravel the complex genetic landscape of diffuse gastric tumors, *Kabbage et al.* investigate a Tunisian family with a potential genetic predisposition to hereditary cancers, including Hereditary Diffuse Gastric Cancer (HDGC) and Lynch Syndrome II (LSII). Utilizing a custom panel for targeted sequencing of DNA repair genes, the researchers identified two significant variants: a rare *MSH2* variant and a novel *FANCD2* variant, both of which are potentially deleterious. This study underscores the importance of targeted clinical surveillance and personalized medicine for patients with suspected genetic predispositions to these cancers. The findings propose the *MSH2* variant as a potential marker for LSII screening, not only in Tunisia but also across the Mediterranean region [15].

Finally, in a groundbreaking study, *Politi et al.* delve into the intriguing genetic phenomenon of SRY-positive females who are healthy despite carrying a rare t(X;Y)(q28;p11.2)(SRY+) translocation. Identified through Non-Invasive Prenatal Testing (NIPT) and confirmed via FISH analysis, these women from three generations exhibit normal female phenotypes, defying the typical expectations of sex chromosome anomalies. The study highlights the role of preferential X-inactivation in suppressing SRY gene expression, explaining their normal sexual development. Additionally, the identification of this translocation as an incidental finding underscores the importance of thorough pre- and post-test counseling. Such counseling is crucial to prepare patients for unexpected discoveries and to guide personal and medical decisions based on the findings [16].

In conclusion, this Special Issue of *Genes* offers a comprehensive overview of the latest advancements in the genetic and molecular basis of inherited disorders. These studies collectively highlight the transformative potential of integrating genetic research with clinical practice, advancing personalized medicine and improving patient outcomes. 

As we continue to unravel the complexities of the human genome, the insights gained from these studies will undoubtedly inspire further research and collaboration, driving progress toward more effective healthcare solutions for individuals with inherited disorders.
